# Fuel-Driven Redox Reactions in Electrolyte-Free Polymer Actuators for Soft Robotics

**DOI:** 10.1021/acsami.3c04883

**Published:** 2023-06-22

**Authors:** Sevketcan Sarikaya, Frank Gardea, Jeffrey T. Auletta, Alex Langrock, Hyun Kim, David M. Mackie, Mohammad Naraghi

**Affiliations:** † Materials Science and Engineering Department, Texas A&M University, College Station, Texas 77843, United States; ‡ Army Research Directorate, Army Research Laboratory South, U.S. Army Combat Capabilities Development Command, College Station, Texas 77843, United States; § Army Research Directorate, Army Research Laboratory, U.S. Army Combat Capabilities Development Command, Adelphi, Maryland 20783, United States; ∥ Army Research Directorate, Army Research Laboratory, U.S. Army Combat Capabilities Development Command, Aberdeen Proving Ground, Maryland 21005, United States; ⊥ Advanced Materials Division, Korea Research Institute of Chemical Technology, Daejeon 34114, South Korea; # Department of Aerospace Engineering, Texas A&M University, College Station, Texas 77843, United States

**Keywords:** artificial muscles, actuators, fuel-powered, catch state, athermal

## Abstract

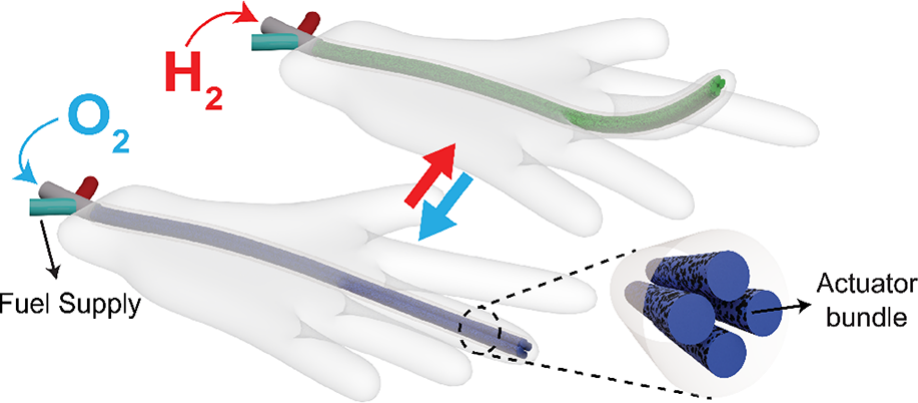

Polymers that undergo shape changes in response to external stimuli can serve as actuators and offer significant potential in a variety of technologies, including biomimetic artificial muscles and soft robotics. Current polymer artificial muscles possess major challenges for various applications as they often require extreme and non-practical actuation conditions. Thus, exploring actuators with new or underutilized stimuli may broaden the application of polymer-based artificial muscles. Here, we introduce an all-solid fuel-powered actuator that contracts and expands when exposed to H_2_ and O_2_ via redox reactions. This actuator demonstrates a fully reversible actuation magnitude of up to 3.8% and achieves a work capacity of 120 J/kg. Unlike traditional chemical actuators, our actuator eliminates the need for electrolytes, electrodes, and the application of external voltage. Moreover, it offers athermal actuation by avoiding the drawbacks of thermal actuators. Remarkably, the actuator maintains its actuated position under load when not stimulated, without consuming energy (i.e., catch state). These fuel-powered fiber actuators were embedded in a soft humanoid hand to demonstrate finger-bending motions. In terms of two main actuation metrics, stress-free contraction strain and blocking stress, the presented artificial muscle outperforms reported polymer redox actuators. The fuel-powered actuator developed in this work creates new avenues for the application of redox polymers in soft robotics and artificial muscles.

## Introduction

The research and development of stimuli-responsive polymers as artificial muscles have grown considerably in the past two decades for a variety of applications, such as for improved robotic mobility and enhanced robot–human interaction.^1^ Polymer artificial muscles have demonstrated mechanical work (i.e., linear contraction and expansion) in response to external stimuli. The commonly used stimuli include heat,^2−4^ electricity,^5,6^ changes in moisture,^7,8^ and pH.^9^ However, these stimuli provide limited practicality for soft actuators and artificial muscles in certain applications. For instance, high-temperature requirements of up to 300 °C and low heating/cooling rates of thermal actuators often prohibit their practical deployment.^10^ On the other hand, voltage-driven polymer actuators (e.g., dielectric polymer actuators) require considerably high voltages and external large footprint, wired power systems. Moreover, pH-responsive polymers typically produce high actuation performances only in extreme pH values, leading to corrosive environments.^9^ These drawbacks present roadblocks for the usage of soft actuators. Therefore, facile triggers to operate these soft actuators are of significant interest for their utilization in various applications.

The growing interest in artificial muscles has expanded to chemically stimulated polymers, inspired by biological muscle systems, which are governed by complex biochemical reactions.^11,12^ A widely known example is electrically conductive redox polymers that show volume changes in response to chemical reactions. This class of redox polymers could directly convert chemical energy into useful mechanical energy for actuation.^11,13−15^ Despite the benefits of the state-of-the-art redox polymer actuators as artificial muscles, their actuation often requires a solid or liquid electrolyte medium with external driving voltages and/or extreme pH conditions.^15−18^ These requirements and inherent system complexity still limit their integration into a practical actuator system. Furthermore, even when twisted and coiled to enhance the actuation, these actuators tend to deliver relatively low actuation magnitudes, generally around a few percent.^19^

Here, we introduce an all-solid, fuel-powered polymer artificial muscle, free of any external electrochemical components. The new actuation mechanism generates motion via fuel-induced redox reactions. We employed catalyst-coated wet-spun polyaniline (PANI), which provides scalable, continuous, conductive, and flexible micro-sized fibers. When exposed to H_2_ or O_2_, the fibers show reversible linear contraction or expansion, respectively. Notably, the fuel-driven actuation mechanism does not require an electrode and electrolyte medium. The fuel-triggered redox reactions and actuation were verified by associated changes in electrical resistance and optical color. The fiber actuators were embedded into a finger of a soft silicone hand and the finger actuated by supplying fuels into an internal chamber.

## Methods

The materials and equipment used in the polymerization, wet spinning, doping, and coating of PANI are summarized in Table S2.

### Polyaniline (PANI) Polymerization

A total of 15.6 g of ammonium persulfate (APS) was mixed with 135.5 mL of deionized (DI) water. Five milliliters of aniline and 7.44 mL of sulfuric acid (H_2_SO_4_) were added to 125 mL of DI water. Next, the APS solution was added to the aniline solution and magnetically stirred in an ice bath. The polymerization was completed overnight. The obtained PANI was washed with ammonium hydroxide (NH_4_OH) and DI water until a pH of 7 was obtained. The PANI powder was dried overnight in an oven at 60 °C.

### Wet Spinning of PANI

A typical preparation procedure to obtain wet-spun PANI fibers is as follows.^16^ PANI powder (0.844 g) and 2-acrylamido-2-methylproprane sulfonic acid (AMPSA) (1.156 g) were mixed by a mortar and pestle. The mixture was added to dichloroacetic acid (DCA) (38 g) and shear-mixed at 7000 rpm for 2 h. The viscous solution was loaded into a syringe with a needle of 21.5G (I.D. 0.5 mm). The syringe was placed onto a syringe pump. The solution was spun into a rotating glass dish (dia. 170 mm) full of acetone. The syringe deposition rate and dish rotation were set to 9 mL/h and 1.2 rpm, respectively. The long, blue-colored PANI fibers were collected from the dish (Figure S1) and copiously washed for a minimum of 2 days. Then, the fibers were dried in an oven at 100 °C for 4 h. The washing and heat treatment turned the color of the fibers into brown. The washed and dried PANI fibers were doped in a 1 M hydrochloric acid (HCl) solution for about 12 h, resulting in conductive, blue-colored fibers.

### Catalyst Coating of Doped PANI Fibers

Platinum (Pt) catalyst ink was prepared as follows. Platinum-on-carbon (Pt/C) particles, having 40 wt % Pt/C (HiSPEC 4000), were mixed with a surfactant (Poloxamer P407) at a Pt/C:P407 weight ratio of 65:35. DI water was used as a solvent, and the final solid-to-solvent ratio was set to 4%. After sonication for 30 min in an ice bath, the catalyst ink solution was further magnetically stirred overnight. The doped fibers were individually dipped into the catalyst ink for about 20 s in such a way that the ink wetted the total surface of each fiber. The fibers were then quickly withdrawn from the catalyst solution. The coated fibers were clipped and dried in an ambient atmosphere. The coated fibers were re-doped in a HCl solution due to the coating procedure leading to some undoping.

### Actuation

Two copper wires were attached to Pt-coated PANI fibers via electrically conductive carbon glue. The wire leads were solely used for monitoring resistivity changes of the sample and to track the generated voltage during actuation. The actuation itself did not require the use of the wires. The sample was placed inside a 5 mL tube (1 mm diameter), which was used as an actuation chamber. The upper end of the wire was tied to the chamber lid. Weights with variable masses were attached to the lower end of the wire with the help of a binder clip. A laser displacement sensor (Panasonic HL-G112) was placed under the binder clip, on which the laser was pointed, to track displacement.

The fuel, H_2_ and O_2_ gases, was supplied by an H-TEC Electrolyzer 230, and N_2_ was provided by a cylindrical gas tank. All gases supplied to the actuation chamber were humidified via an air bubbler to 100% RH. The gas flows were controlled with a flowmeter (Omega FMA). Before the actuation tests, wet N_2_ was supplied to the chamber until the fiber displacement reached a plateau, signifying a fully humidified environment within the chamber. To initiate the actuation cycles, H_2_ was first applied, followed by chamber purging with N_2_, then followed by O_2_, and finally again purged with N_2_. The cycle was repeated for multiple actuation cycles. All gases were applied for 500 s. It is important to note that failure to purge the chamber with N_2_ could lead to burning of the sample, as H_2_ and O_2_ react in the presence of the catalyst.

The actuation of the fibers was measured with the laser sensor, and the contractive axial actuation was calculated as follows:
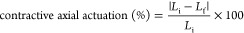
1where *L*_i_ is the initial fiber length and *L*_f_ is the final fiber length after actuation (*L*_f_ < *L*_i_).

The fiber diameter was averaged from cross-sectional SEM images. The applied stresses were calculated based on the diameter of washed, heat-treated, and doped fibers. The artificial muscle work capacity was calculated as:
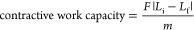
2where *F* is the applied force (weight), and *m* is the total fiber mass.

### Characterization

Voltage and current during fuel-driven actuation of the samples were measured by a digital multimeter (Keithley DAQ6510). The sample ends were connected to the multimeter by copper wires glued with conductive silver paste. Thermogravimetric analysis (TGA) was conducted via a TA Instruments TGA 5500. Scanning electron microscopy (SEM) images of the fibers and their cross-sections were taken via a Tescan LYRA-3. Gel permeation chromatography (GPC) characterization was conducted via a Tosoh GPC using DMF/0.5 wt % LiCl. To prepare the polymer solution, 2 mg of polyaniline powder was dissolved in 2 mL of HPLC grade DMF containing 0.5 wt % LiCl. The solution was passed through a filter prior to the measurement. To characterize the mechanical properties, we conducted tests on wet-spun, washed, dried, and Pt-coated PANI single fibers. These tests were performed according to ASTM C1557 standards using a Deben microtensile stage equipped with a 20 N load cell. We chose a fiber length of 10 mm and a strain rate of 1 mm/min for the tests.

### Redox-Activated Color Change upon Application of Fuels

Aliquots of 1 mL were taken from the previously described PANI/AMPSA/DCA stock solution prepared for wet spinning. Thin films were cast by pipetting 1 mL of the PANI solution onto a glass microscope slide (25 mm × 75 mm), and the solvent was allowed to evaporate in a fume hood. An aqueous catalyst ink was prepared as described in the previous section. A visibly transparent catalyst coating (as observed by the naked eye with a suitable backlight) was obtained by pipetting 20 μL of ink onto the PANI film surface and evenly spreading using a delicate task wipe. After drying, the PANI-Pt glass slide was mounted in a custom-built redox chamber, and dry Ar was purged for 30 s followed by wet H_2_ and O_2_.

### Finger Actuation of a Robotic Soft Hand

A hand mold (male mold) was designed and printed via additive manufacturing. Silicone (Durometer 65A) was cast onto the 3D-printed mold to obtain a silicone female mold. A 1 mm diameter metal rod was placed into the silicone hand mold such that upon removal of the rod after curing, a hollow channel would be created along the finger and palm. A translucent soft silicone rubber (Ecoflex 00-10) was poured into the mold and cured at room temperature (Figure S9a). After curing, the metal rod was carefully removed, resulting in a hollow cylindrical channel spanning from the tip of the finger to the wrist of the hand. This channel would form the chamber for embedding the PANI artificial muscles. A soft and flexible hand with a 73 mm length and 2.5 mm thickness was obtained (Figure S9b). The overall weight of the hand was measured as 2.48 g.

Four wet-spun PANI fibers (∼65 mm in length) were embedded into the middle finger channel of the soft silicone hand. The tip of the finger was punctured, and the embedded fibers were attached to the punctured fingertip via conductive glue (Figure S9c). The fiber ends at the hand wrist were glued to a very thin copper wire, used for circuit connection, via a conductive carbon paste. An electric circuit having a two-colored LED (green and red) was connected to the hand with a 9 V battery. A 26-gauge needle was inserted into the finger channel at the wrist end. Gases (H_2_, N_2_, and O_2_) were supplied to the finger through this needle. After supplying the initial N_2_ (humidifying step), the fiber actuators were initially taut by applying tension on the attached copper wire, thus removing any slack in the fibers.

## Results and Discussion

**Figure 1:**
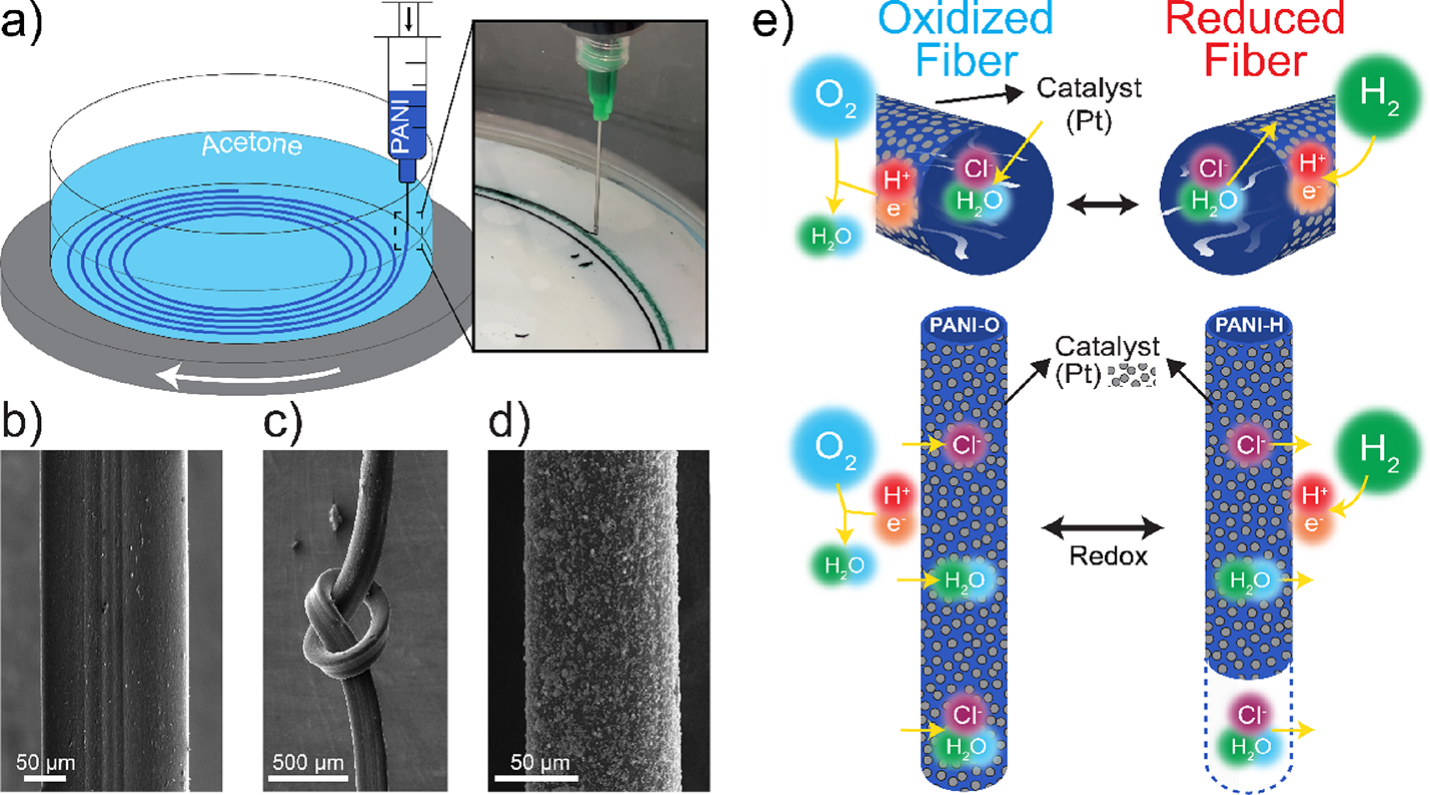
(a) Schematic illustration of the wet-spinning process, along with a photograph during fiber coagulation in acetone, forming long and continuous fibers. SEM images of (b) as-spun, (c) knotted, and (d) Pt-coated PANI fibers (Pt mapping analysis is available in Figure S5). (e) Illustration of the mechanism of reversible fuel-powered actuation. H_2_ chemically reduces Pt-coated PANI, leading to a contraction in fiber length, while O_2_ oxidizes PANI and expands the fiber length, with H_2_O as a byproduct.

The polyaniline used in this study had a weight average molecular weight (*M*_w_) of 23,600 and a polydispersity index (PDI) of 2.47 (Table S3). The fiber actuators were fabricated via the wet-spinning method where the PANI solution (PANI/AMPSA/DCA) was injected into a rotating acetone bath, shown in Figure 1a and Movie S1. The injected PANI solution coagulated inside the bath, forming long and continuous fibers with an average diameter of 144 ± 13 μm (Figure 1b and Figure S1). This wet-spinning method produced flexible and robust fibers that could be knotted, as shown in Figure 1c. The resulting wet-spun fibers were then washed and annealed, removing most of the residual solvents (Figure S2). The washing and annealing process decreased the average diameter to 113 ± 6 μm and turned the fiber color from blue to brown (Figure S3). As washing leads to undoping, the fibers were redoped in 1 M HCl to regain electrical conductivity. Subsequently, the fibers were dip-coated in the Pt catalyst ink, forming a uniform catalyst coating on the fiber surface, as shown in Figure 1d. The cross-sectional SEM images and elemental analysis showing Pt and Cl distribution are available in Figures S4 and S5. The mechanical properties of the fiber were measured as shown in Figure S6, revealing a modulus of 3.77 ± 0.32 GPa, a tensile strength of 109.95 ± 5.47 MPa, and an elongation at break of 6.71 ± 0.25%.

The length expansion and contraction of redox polymers are typically induced by applied voltages or pH changes, which trigger ion and water transfer, facilitated by liquid or solid electrolytes.^20,21^ Our method greatly simplifies this chemical actuation via the use of catalytic fuel-driven reactions. In this method, the Pt-coated fibers were reversibly contracted and expanded by subjecting them to a fuel (H_2_) and oxidizer (O_2_), respectively (Figure 1e). This actuation response occurred due to the reduction and oxidation states of PANI (Figure S7), dictating counterion and water migrations.^16,22,23^ When the catalyst-coated fibers were exposed to H_2_, the catalyst oxidized H_2_ (H_2_→2H^+^ + 2e^–^) and the surface became H^+^-rich. PANI was reduced by anion (Cl^–^) de-insertion accompanied by water migration, leading to a decrease in volume and length. In the complementary half cycle of actuation, O_2_ was supplied to the actuator, oxidizing PANI by extraction of H^+^ from the surface (O_2_ + 4H^+^ + 4e^–^→2H_2_O). The removal of surface protons led to insertion of anions, thus drawing water into the polymer backbone, leading to an increase in actuator volume and length.

**Figure 2:**
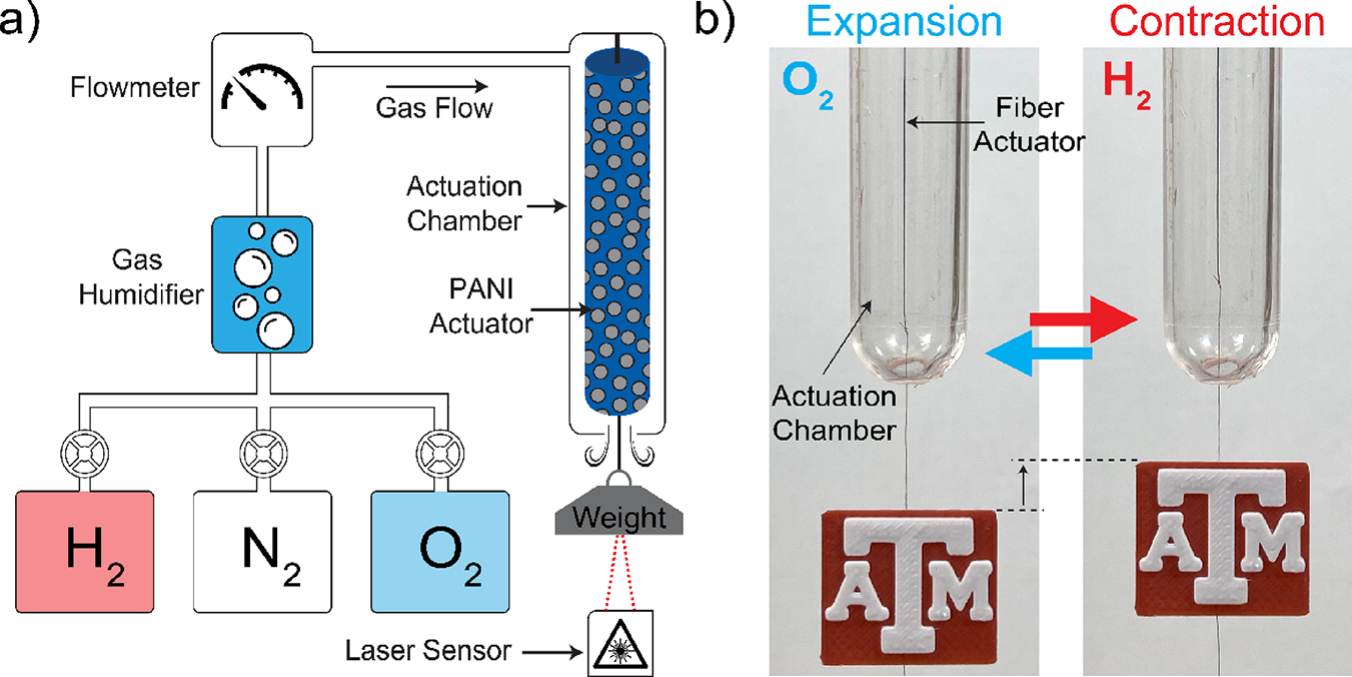
(a) Illustration of the test setup used in fuel-driven actuation. The bubbler set the gases to 100% humidity. The flowmeter controlled the amount of gas supplied. The laser sensor measured the displacement of actuators. (b) Photographs of a single fiber actuator in an actuation chamber. The fiber was cycled between reduced (with H_2_) and oxidized (with O_2_) states of the polymer. The 9 cm-long fiber gave a 3.8% contraction under 0.75 MPa stress.

Reversible linear actuation was conducted via a customized setup shown in Figure 2a, consisting of an actuation chamber in which the fiber actuators were placed. The H_2_ and O_2_ gases supplied to the chamber contracted and expanded the fiber actuator length, respectively, and N_2_ purged the chamber after each contraction or expansion cycle. Corresponding actuation photos are shown in Figure 2b, where the fuel-driven actuator shows a ∼3.8% actuation under 0.75 MPa stress. Notably, unlike the traditional chemically stimulated soft actuators, the actuation mechanism demonstrated here enables the direct usage of fuels without the use of any type of liquid or solid electrolyte. No coupling counter electrode was required, and no temperature change was observed during actuation cycles (Figure S8). It is likely that the reactions generate/absorb only a small amount of heat, which is quickly dissipated to the environment, insufficient to noticeably change the actuator’s temperature.

**Figure 3:**
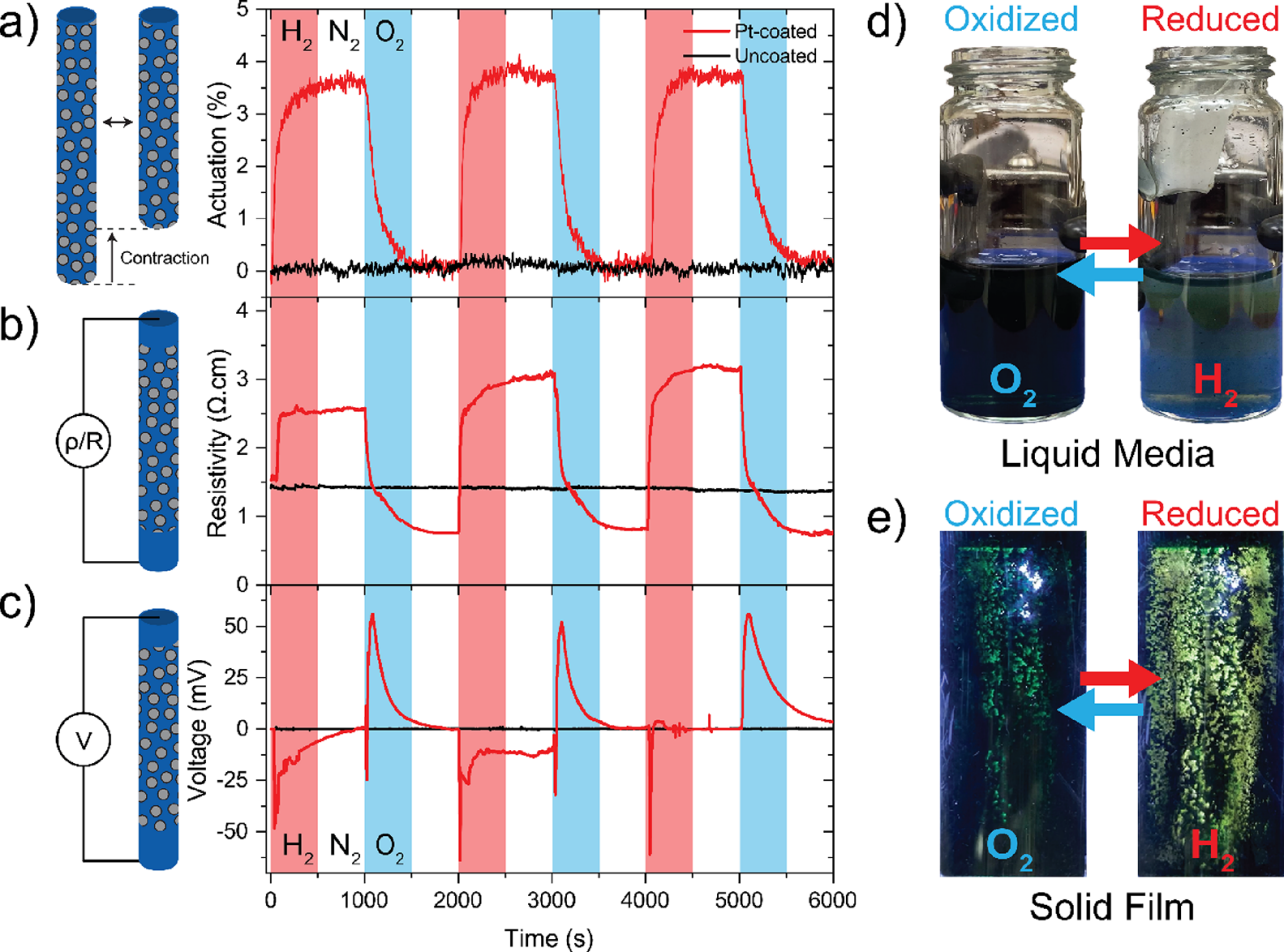
(a) Linear contractive tensile stroke under 0.75 MPa and corresponding (b) resistivity and (c) voltage change with response to actuation via fuel gases (H_2_ and O_2_) and purging N_2_. Fuel-stimulated color change of (d) PANI/Pt in HCl solution and (e) Pt-coated doped PANI thin film.

The fuel-driven actuation was only enabled in the presence of the Pt catalyst coated on the fibers. As shown in Figure 3a, uncoated fibers showed no actuation, while Pt-coated fibers contracted in length by 3.8%. These results suggest that the fuel interacting with the catalyst and polymer (i.e., the redox reaction) was the only energy source for actuation. Moreover, the change in redox states (reduction ↔ oxidation) was also inferred from the changes in electrical resistivity (Figure 3b) and generated voltage (Figure 3c), which correlated and perfectly synced with the actuation response. Upon reduction or oxidation, the change in PANI conductivity arises from the change in ion content in the polymer backbone, affecting water retention properties and leading to volume and length changes.^21,24,25^ As seen in Figure 3b, the resistivity increased with H_2_ application and decreased with O_2_. Prior to any reaction, the resistivity of the polymer fiber was about 1.5 Ω·cm and the supply of H_2_ (reduction) doubled the resistivity to 3 Ω·cm. On the other hand, O_2_, or oxidation of the actuator, decreased the polymer resistivity to 0.8 Ω·cm and made the polymer more hydrophilic due to charge compensation. Thus, the actuator expanded and increased its volume and length via water absorption from the environment. The 275% resistivity change in response to fuel was higher than the resistivity change of 26% due to the change in humidity (Table S4). This provides further evidence in support of the fuel-driven redox reactions. Similarly, H_2_ and O_2_ demonstrated opposite-sign voltage changes, confirming that reactions occurred in opposite directions, as shown in Figure 3d. It is also important to note that when we tried to operate the actuator under fully dried conditions (0% humidity), the actuation did not occur. Therefore, we conclude that the absence of water molecules impedes ion migration and thus prevents the actuation from taking place.

Redox states of PANI yield different colors resulting from a change in electronic and chemical structure. The oxidation of PANI results in a dark blue color, while reduction renders a green color.^26,27^ To verify redox reactions induced by fuels, we investigated redox derivative color changes in our catalyst/PANI systems. In Figure 3d, PANI/Pt in 1 M HCl solution was reduced with H_2_, leading to a light green color. Oxidation of the solution with O_2_ switched the color to dark blue. To verify the redox reactions in solid PANI form, a thin film of PANI coated with the Pt catalyst was exposed to fuels (H_2_ and O_2_). Similar to PANI in solution, hydrogeneration (reduction with H_2_) turned the film into a green color, whereas oxidation with O_2_ resulted in a dark blue color (Figure 3e). The distinct color changes clearly show and confirm the altered oxidation state of PANI/Pt by the supplied fuels.

**Figure 4:**
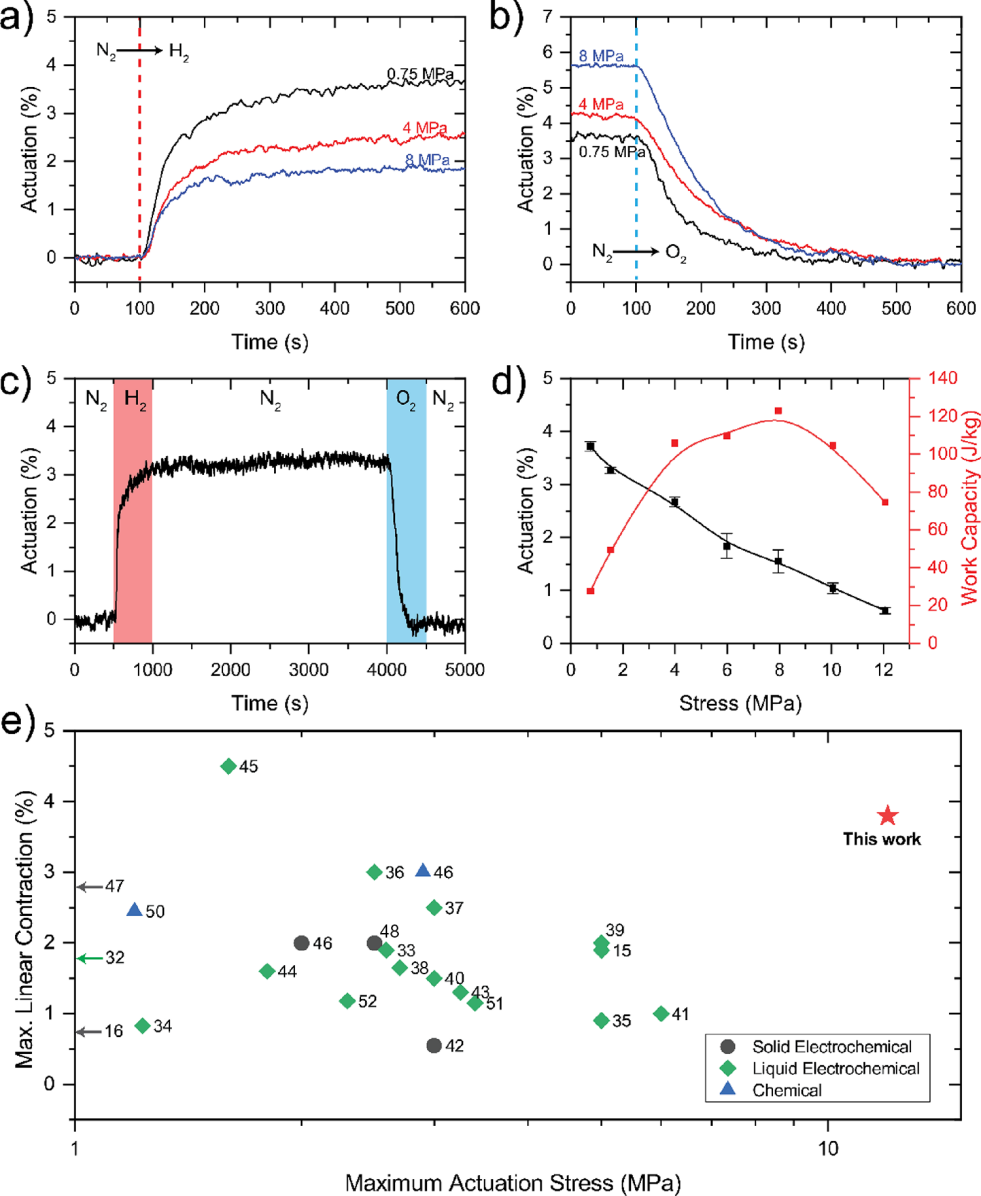
(a) Contraction versus time behavior during the N_2_-to-H_2_ transition and (b) expansion versus time behavior during N_2_-to-O_2_ transition for fibers with three different applied stresses (0.75, 4, and 8 MPa. (c) Energy-free catch state where the actuator maintained the contracted position without the stimulus under 1 MPa stress. (d) Actuation stroke and work capacity as a function of applied stress. (e) Maximum linear contraction and actuation stress comparison of literature-reported redox polymer actuators.^15,16,32−52^ Data and sources are available in Table S5.

The contraction/expansion magnitude and rate of the fuel-stimulated actuator were also functions of the applied stress. During contraction (reduction, N_2_ → H_2_) in Figure 4a, the increased actuation stress also decreased the maximum actuation obtained. Under a stress of 0.75 MPa, the actuator provided 3.8% contraction. During this actuation, it demonstrated contraction and expansion rates of 3.52%/min and 2.38%/min, respectively, within the first 30 s. However, as the applied stress was incrementally increased to 4 and 8 MPa, the degree of actuator contraction correspondingly decreased to 2.5 and 1.9%, respectively. This increased stress also led to reduced contraction rates, dropping to 1.78%/min and 1.64%/min under the stresses of 4 and 8 MPa. As an example, the actuator under 0.75 MPa achieved a 1% contraction within 20 s, whereas under stresses of 4 and 8 MPa, the same 1% contraction level was only reached after 35 and 40 s, respectively. These actuation rates surpass other fuel-driven and electrochemical actuators,^28,29^ without the need to actuate in liquid media. On the other hand, during the expansion (oxidation, N_2_ → O_2_) in Figure 4b, a 0.75 MPa applied stress elongated the fiber by 3.8%, showing no creep. As the fiber experienced larger stresses (e.g., 8 MPa), it demonstrated larger expansions due to the combined effect of axial stretching and length expansion.

This fuel-driven actuator can maintain its actuated state even after the fuel is switched off. In this catch or lock-up state, the actuator position is conserved without any energy consumption. This highly advantageous feature is observed in some biological muscles, but it is uncommon among artificial muscles.^30^Figure 4c demonstrates this behavior in our fuel-stimulated artificial muscle where the actuator retained its contracted position for 50 min under 1 MPa load without creep. Furthermore, an extended period in the catch state had no effect on actuation properties as the actuator was able to expand/contract repeatedly. This observed catch-state feature outpaces most polymer actuators reported, such as thermal actuators that require heat supply to maintain actuation temperature and position.

A peak work capacity of 120 J/kg was achieved under 8 MPa stress, which is 15 times higher than typical human muscles (∼7.7 J/kg).^31^ Based on the highest working capacity range obtained, the optimum actuation stress range was found to be 4–8 MPa. The maximum stress that the actuator lifted was 12 MPa, corresponding to a 75 J/kg work capacity (Figure 4d). To compare our actuation performance with other redox-driven polymers, a graph indicating maximum contraction and actuation stress is provided in Figure 4e. For a better comparison, the graph includes only soft materials that provide linear actuation. Temperature-driven polymers are excluded as they require and release considerable heat in return for larger actuation. From the graph, our fuel-powered actuator outperforms other redox-driven polymer actuators in terms of actuation strain and stress. Apart from actuation performance, our actuator offers more practical integration to applications since it does not require highly acidic actuation media, externally applied voltages, and/or changes in temperature.

**Figure 5:**
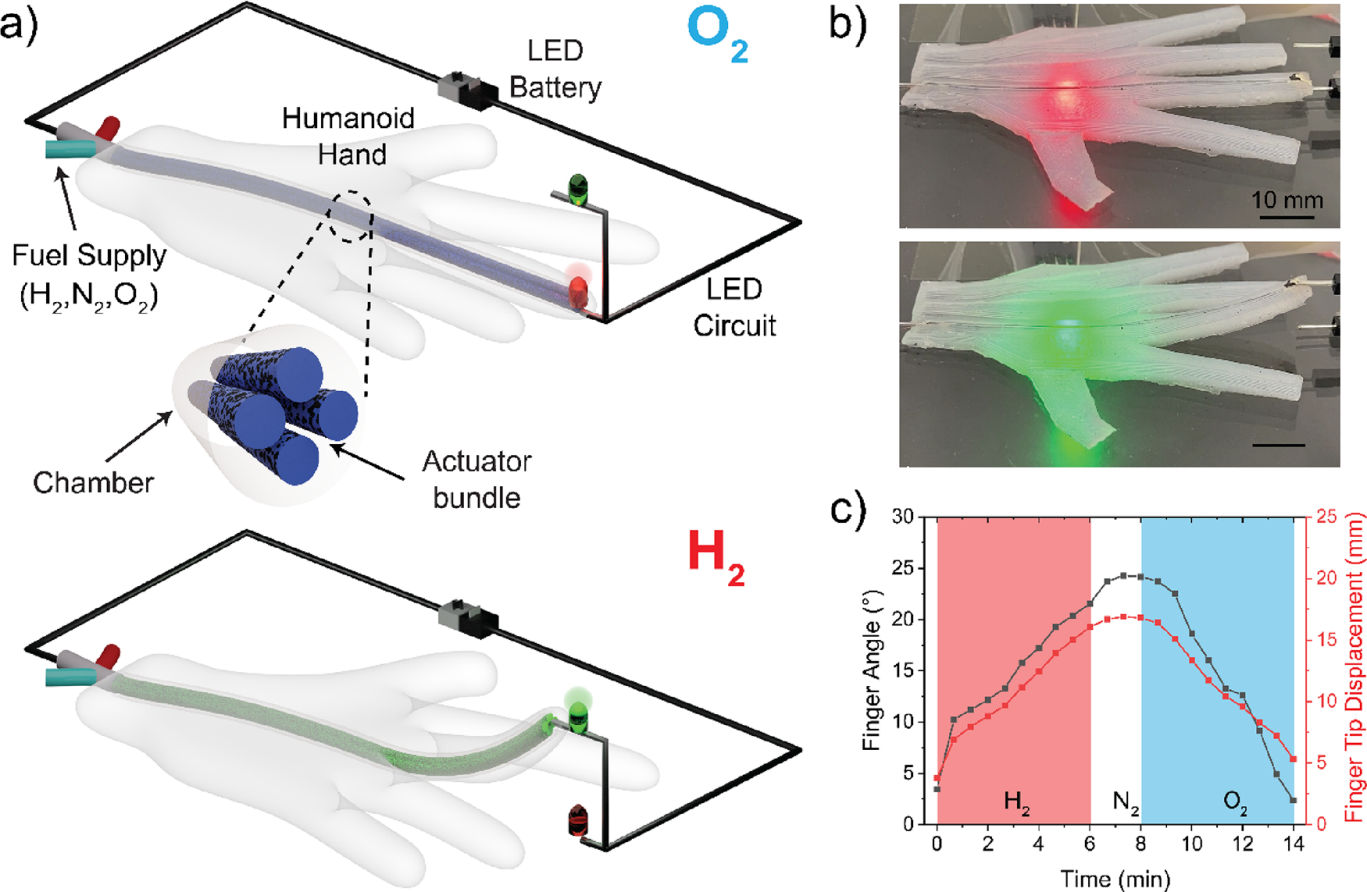
(a) Illustration of the fuel-powered soft hand of which the inner actuation channel encloses conductive fiber actuators connected to the LED circuits. As the fibers contract in length with H_2_, the finger bends upward. As the oxidized fibers with O_2_ expand in length, the finger returns to its initial position. The two-way finger switch circuit can light green (ON) and red (OFF) LEDs via intrinsically conductive artificial muscles. (b) Pictures of the humanoid hand switching the LED circuit by finger motion driven by embedded artificial muscles. The supplementary video is available as Movie S2. (c) Change in finger angle and tip displacement with respect to time.

The fuel-driven actuator can be integrated into form factors in various applications. Inspired by biological systems, which contain chemically driven muscles and electrically conductive nerves, we embedded the fuel-driven fibers into a humanoid soft hand, fabricated from a commercially available skin-safe silicone. As shown in Figure 5a, this humanoid hand consisted of a cylindrical inner channel (73 mm in length) routing through the palm and finger. As shown in Figure S9, the actuator fibers were embedded into the inner channel, which facilitated actuation by acting as a gas chamber. The actuation gases were supplied from the wrist through the middle finger channel.

The finger motions resulted from the linear contraction or expansion of the fibers placed at an offset position from the neutral axis of the finger. The H_2_ application initiated the linear contraction of the embedded fibers in the hand. The fiber contraction was simultaneously converted to finger-bending motion. The finger bent upward with a maximum bending angle of 24°, which is higher by ∼7° than a human metacarpophalangeal (MCP) joint.^53^ The total perpendicular displacement of the finger was 13 mm (Figure 5c). After purging the actuator channel with N_2_, O_2_ returned the finger to its initial natural position.

This humanoid hand can serve as a two-way switch by leveraging both the electrical conductivity and mechanical motion of the fibers. As illustrated in Figure 5a, the hand was connected to a two-color LED and two-way circuit. The electrically conductive fibers formed a completed circuit upon contact with one of two LEDs. In a straight, unactuated state, the conductive actuator fibers closed the red LED circuit and established the “OFF” position (OFF → red light). With the supply of H_2_, the finger actuation opened the circuit and turned off the red LED. Upon contact of the fingertip with the upper green LED, the second circuit was closed, lighting a green LED and establishing an “ON” position (ON → green light). The actuation of the finger was then reversed and returned to an “OFF” position. The pictures and video of the ON and OFF finger positions are seen in Figure 5b and Movie S2.

This demonstration shows the potential of these actuators as multi-purpose components in soft robotics. Not only can these fibers provide chemically powered actuation, but they also have the potential to introduce electrical energy transmission throughout a robotic platform, thus simultaneously imparting other capabilities, such as sensing.

## Conclusions

We demonstrated a fuel-powered, all-solid polymer actuator that provides linear motion via H_2_ and O_2_ gases. The actuation mechanism is free of electrolyte and temperature changes. Tensile stroke arises from switching redox states of the PANI fiber upon exposure to gaseous fuel. This fuel-driven redox actuator can maintain a contracted position without any energy expenditure (catch state) and deliver 3.8% actuation strain and 120 J/kg work capacity. We also demonstrated the utility of this method in robotic applications and deployed the fiber actuators into a humanoid hand. This polymer artificial muscle-mechanized hand shows finger motions powered by the actuation fuels. The finger reached a bending angle of 24°, outpacing an equivalent human joint. The polymeric nature of this system could facilitate direct robot–human interactions while the fuel-powered redox actuation is in action.

## Supporting Information Available

Movie 1: Wet spinning of PANI (MP4)
Movie 2: Actuation of the humanoid hand (MP4)
Figure S1: photographs of the fibers in a coagulation bath during the wet-spinning process of PANI; Figure S2: TGA results of PANI powder after polymerization, as-spun fiber, and washed and annealed fiber; Figure S3: optical images of wet-spun fibers: knotted, as-spun, washed, and heat-treated at 100 °C; Figure S4: SEM images of the cross section of as-spun PANI fiber and catalyst-coated PANI fiber and higher-magnification horizontal and vertical SEM images of the coating/fiber interfaces; Figure S5: SEM and EDS characterization of the PANI fiber; Figure S6: stress–strain curves of wet-spun, washed, dried, and Pt-coated PANI single fibers; Table S1: mechanical properties of wet-spun, washed, dried, and Pt-coated PANI single fibers; Figure S7: chemical structure of oxidized PANI and reduced PANI; Figure S8: photograph of the actuation chamber used for thermal imaging and thermal imaging during actuation showing no temperature change in the actuator and chamber; Figure S9: fabrication of silicone hand with embedded fiber actuators; Table S2: materials and components used in the preparation of actuator fibers including PANI polymerization, fiber wet spinning, doping, and Pt coating; Table S3: molecular weights of polyaniline obtained by gel permeation chromatography (GPC); Table S4: resistivity change of PANI fiber under wet (98% RH) and dry (2% RH) N_2_; Table S5: maximum contraction strain and actuation stress of redox polymer actuators reported in the literature (PDF)
