# Supplementary material for: Fuel-Driven Redox Reactions in Electrolyte-Free Polymer Actuators for Soft Robotics
Source: ACS Appl Mater Interfaces. 2023 Jun 22;15(26):31803–11. doi: 10.1021/acsami.3c04883 (PMC10862377; doi:10.1021/acsami.3c04883)
Supplement: Supplementary file 3 — am3c04883_si_003.pdf [file am3c04883_si_003.pdf]

Supporting Information for

Fuel-Driven Redox-Reactions in Electrolyte-free Polymer Actuators for Soft Robotics

Sevketcan Sarikaya[1], Frank Gardea[2], Jeffrey T. Auletta[3], Alex Langrock[4], Hyun Kim[3,5], David M. Mackie[3], Mohammad Naraghi[1,6,*]

1. Materials Science and Engineering Department, Texas A&M University, College Station, TX 77843, USA
2. Army Research Directorate, U.S. Army Combat Capabilities Development Command, Army Research Laboratory South, College Station, TX 77843, USA
3. Army Research Directorate, U.S. Army Combat Capabilities Development Command, Army Research Laboratory, Adelphi, MD 20783, USA
4. Army Research Directorate, U.S. Army Combat Capabilities Development Command, Army Research Laboratory, Aberdeen Proving Ground, MD 21005, USA
5. Advanced Materials Division, Korea Research Institute of Chemical Technology, Daejeon, 34114, South Korea
6. Department of Aerospace Engineering, Texas A&M University, College Station, TX 77843, USA

* Corresponding author, email: naraghi@tamu.edu

The PDF file includes:

Figs. S1 to S9

Table S1 and S5

References

Other Supplementary Material for this manuscript includes the following:

Movies S1 and S2

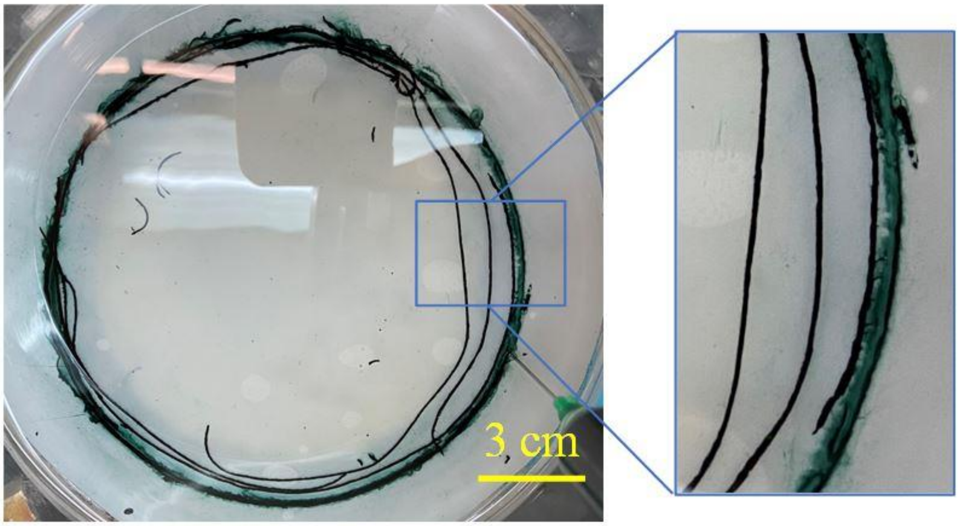

Figure S1. Photographs of the fibers in a coagulation bath during the wet spinning process of PANI.

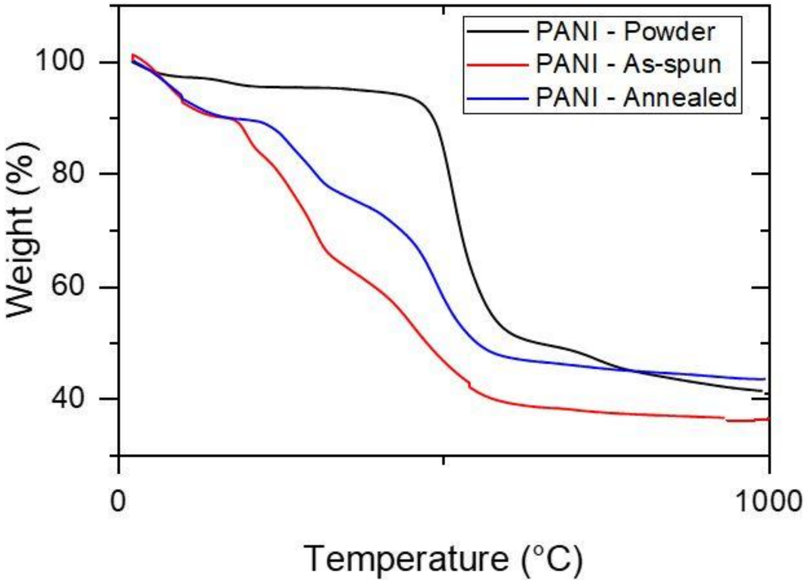

Figure S2. TGA results of PANI powder after polymerization, as-spun fiber, and washed and annealed fiber.

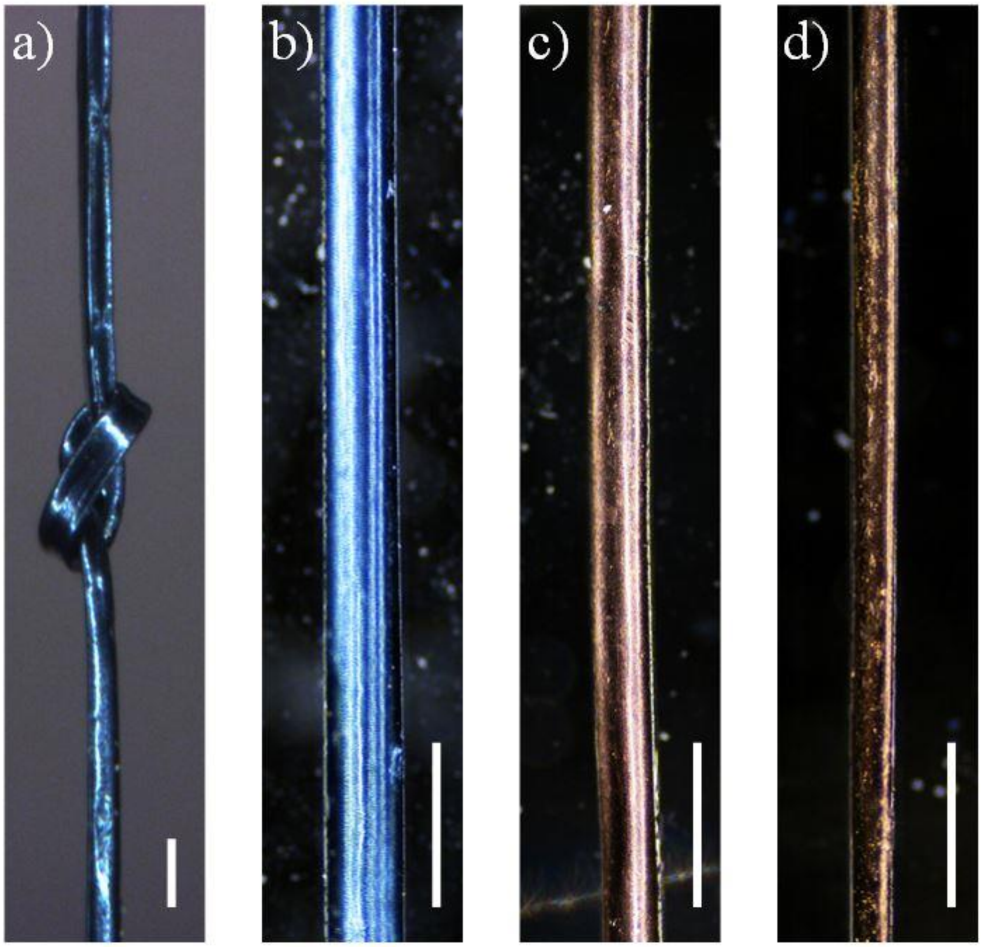

Figure S3. Optical images of wet-spun fibers a) knotted b) as-spun c) washed and d) heat treated at 100 °C. Scale bars represent 500 μm.

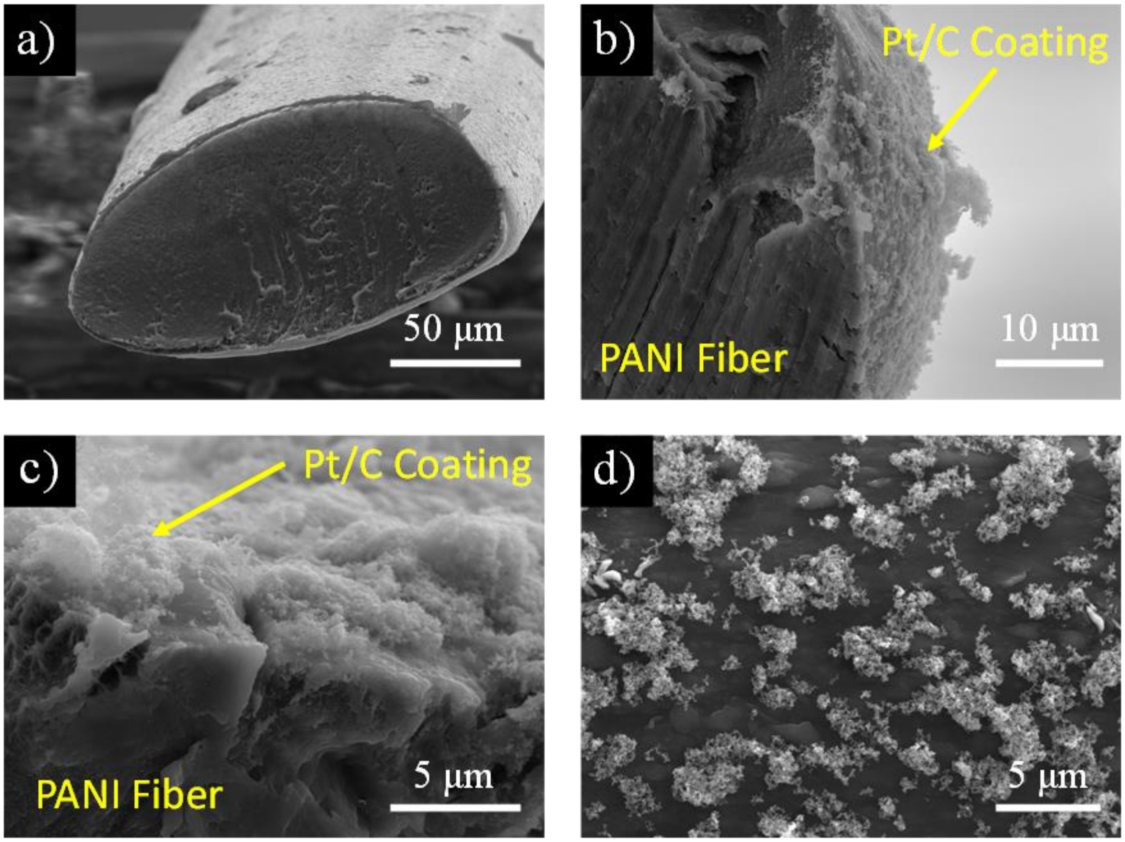

Figure S4. SEM images of the cross-section of a) as-spun PANI fiber and b) catalyst-coated PANI fiber. A higher magnification c) horizontal and d) vertical SEM images of the coating/fiber interfaces

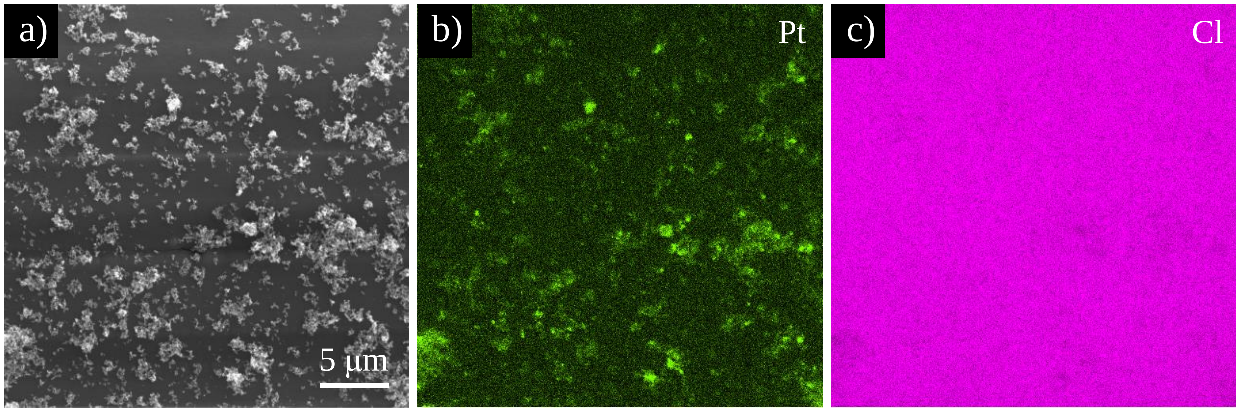

Figure S5. SEM and EDS characterization of the PANI fiber. a) SEM image revealing the surface morphology of the fiber. b) Energy Dispersive X-ray Spectroscopy (EDS) map showing the dispersion of Pt on the fiber surface, indicating the distribution of the catalyst. c) EDS map presenting the distribution of Chlorine (Cl) on the fiber surface, associated with the ion migration during actuation

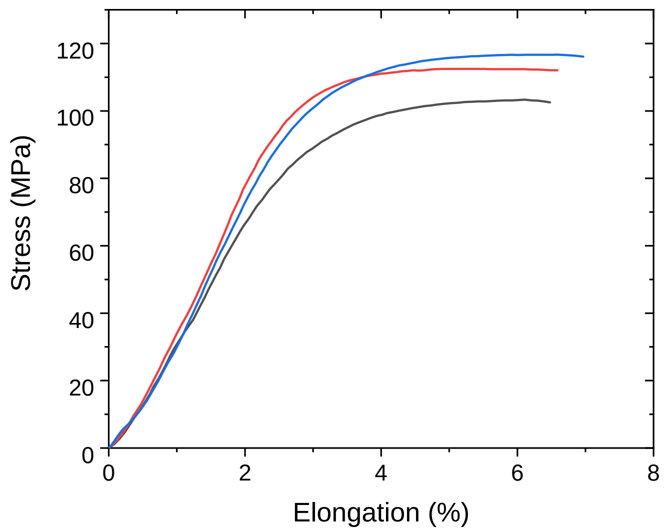

Figure S6. Stress-strain curves of wet-spun, washed, dried, and Pt-coated PANI single fibers. Modulus, strength, and elongation at break were calculated and summarized in Table S1 below.

Table S1. Mechanical properties of wet-spun, washed, dried, and Pt-coated PANI single fibers

**Modulus (GPa)**	**Strength (MPa)**	**Elongation at Break (%)**
3.77 ± 0.32	109.95 ± 5.47	6.71 ± 0.25

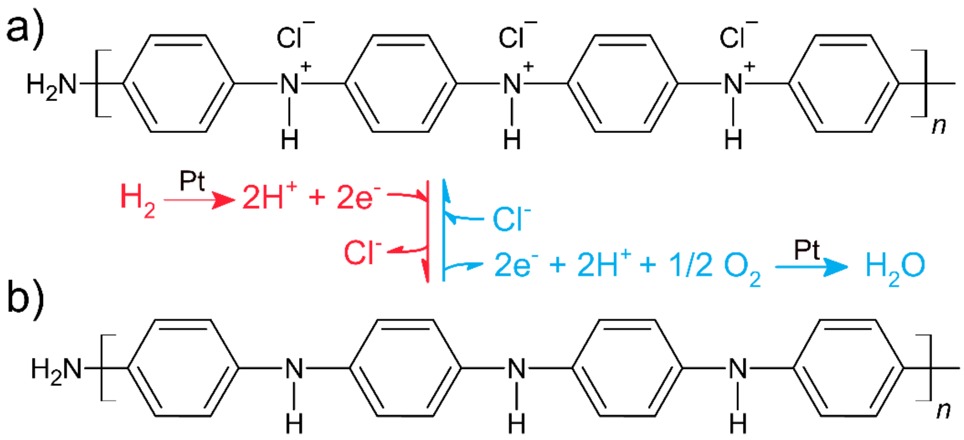

Figure S7. Chemical structure of a) oxidized PANI and b) reduced PANI. The chemical reaction shows reduction (red) where PANI loses an anion via H_2 and oxidation (blue) where an anion is inserted into PANI via O_2.

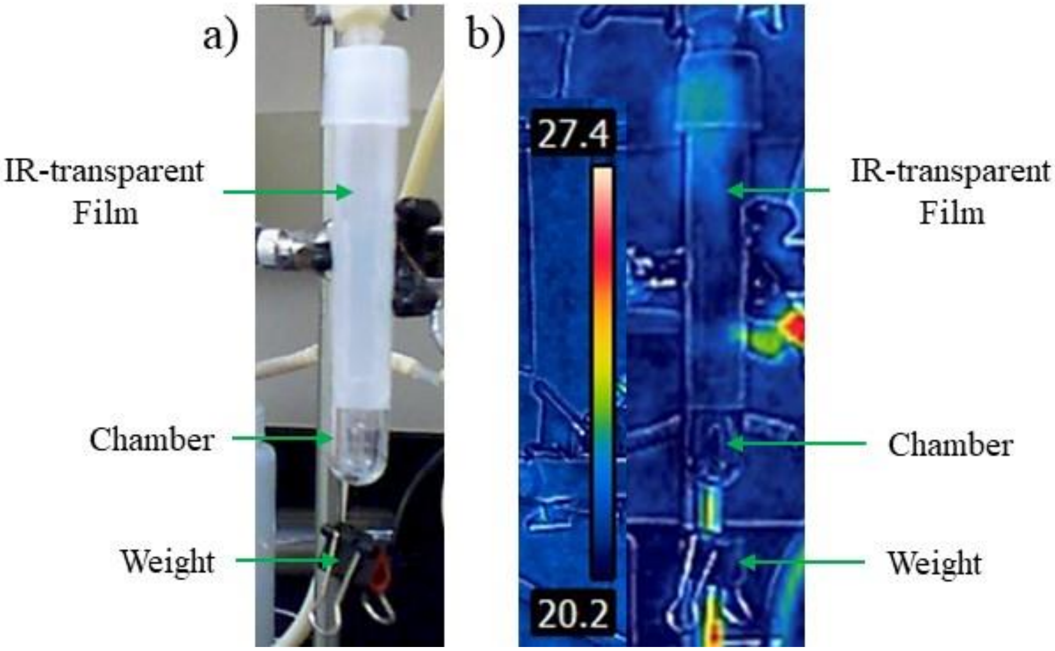

Figure S8. a) Photograph of the actuation chamber used for thermal imaging. The front face of the actuation chamber was covered with an IR visible film to detect IR signals with a thermal camera. b) Thermal imaging during actuation showing no temperature change in the actuator and chamber.

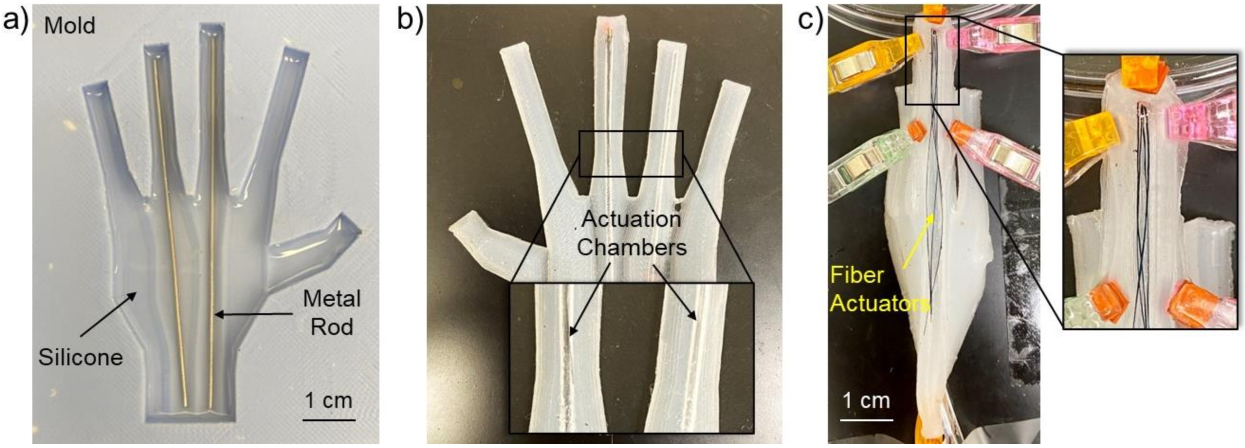

Figure S9. Fabrication of silicone hand with embedded fiber actuators. a) Metal rods were placed in the mold to create inner channels for fiber placement. b) Cured silicone hand showing chambers resulting from removal of rods. c) Placement of the fiber actuators into the inner chamber of the hand. The fibers were glued at the fingertip.

Table S2. The materials and components used in the preparation of actuator fibers including PANI polymerization, fiber wet-spinning, doping, and Pt-coating.

**PANI Polymerization**	
Ammonium persulfate	15.6 g
Sulfuric Acid	7.44 ml
Ammonium hydroxide	-
DI water	-
**Wet-Spinning Solution & Components**	
Powdered PANI	0.844 g
2-acrylamido-2-methylproprane sulfonic acid	1.156 g
Dichloroacetic acid	38 g
Syringe (10 ml)	-
Syringe Needle (21.5 G)	-
Syringe Pump	-
**Proton Doping (1M HCl)**	
Hydrochloric acid (37% w/w)	8.212 mL
DI Water	91.788 mL
**Catalyst Coating**	
Pt/C (HiSPEC 4000)	0.52 g
Poloxamer P407	0.28 g
DI Water	19.2 g

Table S3. Molecular weights of Polyaniline obtained by gel permeation chromatography (GPC)

**M_n**	9555
**M_w**	23607
**M_z**	48747
**M_w/ M_n (Polydispersity Index)**	2.471

Table S4. Resistivity change of PANI fiber under wet (98% RH) and dry (2% RH) N_2.

	**Dry Resistivity (Ω·cm)**	**Wet Resistivity (Ω·cm)**
**PANI (undoped)**	Nonconductive	Nonconductive
**HCl-doped PANI**	1.61	1.27

Table S5. Maximum contraction strain and actuation stress of redox polymer actuators reported in the literature.

Max. Contraction (%)	Max. Actuation Stress (MPa)	Actuation Mechanism	Actuation Media	Ref
3.8	12	Fuel	Gas	This Work
0.05	Not reported	Fuel	Liquid	1
0.9	0.9	Electrochemical	Air	2
0.55	3	Electrochemical	Air	3
NR	5.1	Electrochemical	Air	4
2	2	Electrochemical	Air	5
3	0.23	Electrochemical	Air	6
2	2.5	Electrochemical	Air	7
3	2.9	Chemical	Liquid	8
2.45	0.12	Chemical	Liquid	9
2	Not reported	Chemical	Liquid	10
1.15	3.4	Electrochemical	Liquid	11
1.18	2.3	Electrochemical	Liquid	12
1.9	5	Electrochemical	Liquid	13
1.95	0.2	Electrochemical	Liquid	14
1.9	2.59	Electrochemical	Liquid	15
0.83	1.23	Electrochemical	Liquid	16
0.9	5	Electrochemical	Liquid	17
3	2.5	Electrochemical	Liquid	18
2.5	3	Electrochemical	Liquid	19
1.65	2.7	Electrochemical	Liquid	20
2	5	Electrochemical	Liquid	21
1.5	3	Electrochemical	Liquid	22
1	6	Electrochemical	Liquid	23
1.3	3.25	Electrochemical	Liquid	24
1.6	1.8	Electrochemical	Liquid	25
4.5	1.6	Electrochemical	Liquid	26

References

(1) Ebron, V. H.; Yang, Z.; Seyer, D. J.; Kozlov, M. E.; Oh, J.; Xie, H.; Razal, J.; Hall, L. J.; Ferraris, J. P.; MacDiarmid, A. G.; Baughman, R. H. Fuel-Powered Artificial Muscles. *Science* **2006**, *311* (5767), 1580–1583. https://doi.org/10.1126/science.1120182.

(2) Lu, W.; Smela, E.; Adams, P.; Zuccarello, G.; Mattes, B. R. Development of Solid-in-Hollow Electrochemical Linear Actuators Using Highly Conductive Polyaniline. *Chem. Mater.* **2004**, *16* (9), 1615–1621. https://doi.org/10.1021/cm030159l.

(3) Mazzoldi, A.; Degl'Innocenti, C.; Michelucci, M.; De Rossi, D. Actuative Properties of Polyaniline Fibers under Electrochemical Stimulation. *Mater. Sci. Eng. C* **1998**, *6* (1), 65–72. https://doi.org/10.1016/S0928-4931(98)00036-8.

(4) Hutchison, A. .; Lewis, T. .; Moulton, S. .; Spinks, G. .; Wallace, G. . Development of Polypyrrole-Based Electromechanical Actuators. *Synth. Met.* **2000**, *113* (1–2), 121–127. https://doi.org/10.1016/S0379-6779(00)00190-9.

(5) Madden, J. D.; Cush, R. A.; Kanigan, T. S.; Brenan, C. J.; Hunter, I. W. Encapsulated Polypyrrole Actuators. *Synth. Met.* **1999**, *105* (1), 61–64. https://doi.org/10.1016/S0379-6779(99)00034-X.

(6) Plesse, C.; Vidal, F.; Teyssié, D.; Chevrot, C. Conducting Polymer Artificial Muscle Fibres: Toward an Open Air Linear Actuation. *Chem. Commun.* **2010**, *46* (17), 2910. https://doi.org/10.1039/c001289k.

(7) Fannir, A.; Temmer, R.; Nguyen, G. T. M.; Cadiergues, L.; Laurent, E.; Madden, J. D. W.; Vidal, F.; Plesse, C. Linear Artificial Muscle Based on Ionic Electroactive Polymer: A

Rational Design for Open-Air and Vacuum Actuation. *Adv. Mater. Technol.* **2019**, *4* (2), 1–8. https://doi.org/10.1002/admt.201800519.

(8) Herod, T. E.; Schlenoff, J. B. Doping-Induced Strain in Polyaniline: Stretchoelectrochemistry. *Chem. Mater.* **1993**, *5* (7), 951–955. https://doi.org/10.1021/cm00031a013.

(9) Spinks, G. M.; Shin, S. R.; Wallace, G. G.; Whitten, P. G.; Kim, I. Y.; Kim, S. I.; Kim, S. J. A Novel "Dual Mode" Actuation in Chitosan/Polyaniline/Carbon Nanotube Fibers. *Sensors Actuators, B Chem.* **2007**, *121* (2), 616–621. https://doi.org/10.1016/j.snb.2006.04.103.

(10) Küttel, C.; Stemmer, A.; Wei, X. Strain Response of Polypyrrole Actuators Induced by Redox Agents in Solution. *Sensors Actuators, B Chem.* **2009**, *141* (2), 478–484. https://doi.org/10.1016/j.snb.2009.06.044.

(11) Smela, E.; Mattes, B. R. Polyaniline Actuators: Part 2. PANI(AMPS) in Methanesulfonic Acid. *Synth. Met.* **2005**, *151* (1), 43–48. https://doi.org/10.1016/j.synthmet.2005.02.017.

(12) Qi, B.; Lu, W.; Mattes, B. R. Strain and Energy Efficiency of Polyaniline Fiber Electrochemical Actuators in Aqueous Electrolytes. *J. Phys. Chem. B* **2004**, *108* (20), 6222–6227. https://doi.org/10.1021/jp031092s.

(13) Smela, E.; Lu, W.; Mattes, B. R. Polyaniline Actuators: Part 1. PANI(AMPS) in HCl. *Synth. Met.* **2005**, *151* (1), 25–42. https://doi.org/10.1016/j.synthmet.2005.03.009.

(14) Takashima, W.; Fukui, M.; Kaneko, M.; Kaneto, K. Electrochemomechanical Deformation of Polyaniline Films. *Jpn. J. Appl. Phys.* **1995**, *34* (7S), 3786–3789.

https://doi.org/10.1143/JJAP.34.3786.

(15) Spinks, G. M.; Campbell, T. E.; Wallace, G. G. Force Generation from Polypyrrole Actuators. *Smart Mater. Struct.* **2005**, *14* (2), 406–412. https://doi.org/10.1088/0964-1726/14/2/015.

(16) Tahhan, M.; Truong, V.-T.; Spinks, G. M.; Wallace, G. G. Carbon Nanotube and Polyaniline Composite Actuators*. *Smart Mater. Struct.* **2003**, *12* (4), 626–632. https://doi.org/10.1088/0964-1726/12/4/313.

(17) Mottaghitalab, V.; Xi, B.; Spinks, G. M.; Wallace, G. G. Polyaniline Fibres Containing Single Walled Carbon Nanotubes: Enhanced Performance Artificial Muscles. *Synth. Met.* **2006**, *156* (11–13), 796–803. https://doi.org/10.1016/j.synthmet.2006.03.016.

(18) Ismail, Y. A.; Shin, M. K.; Kim, S. J. A Nanofibrous Hydrogel Templated Electrochemical Actuator: From Single Mat to a Rolled-up Structure. *Sensors Actuators, B Chem.* **2009**, *136* (2), 438–443. https://doi.org/10.1016/j.snb.2008.10.052.

(19) Della Santa, A.; De Rossi, D.; Mazzoldi, A. Performance and Work Capacity of a Polypyrrole Conducting Polymer Linear Actuator. *Synth. Met.* **1997**, *90* (2), 93–100. https://doi.org/10.1016/S0379-6779(97)81256-8.

(20) Gu, B. K.; Ismail, Y. A.; Spinks, G. M.; Kim, S. I.; So, I.; Kim, S. J. A Linear Actuation of Polymeric Nanofibrous Bundle for Artificial Muscles. *Chem. Mater.* **2009**, *21* (3), 511–515. https://doi.org/10.1021/cm802377d.

(21) Sendai, T.; Suematsu, H.; Kaneto, K. Anisotropic Strain and Memory Effect in Electrochemomechanical Strain of Polypyrrole Films under High Tensile Stresses. *Jpn. J.*

Appl. Phys. **2009**, *48* (5), 0515061–0515064. https://doi.org/10.1143/JJAP.48.051506.

(22) Temmer, R.; Must, I.; Kaasik, F.; Aabloo, A.; Tamm, T. Combined Chemical and Electrochemical Synthesis Methods for Metal-Free Polypyrrole Actuators. *Sensors Actuators, B Chem.* **2012**, *166–167*, 411–418. https://doi.org/10.1016/j.snb.2012.01.075.

(23) Pytel, R. Z.; Thomas, E. L.; Hunter, I. W. In Situ Observation of Dynamic Elastic Modulus in Polypyrrole Actuators. *Polymer (Guildf).* **2008**, *49* (8), 2008–2013. https://doi.org/10.1016/j.polymer.2008.01.053.

(24) Della Santa, A.; De Rossi, D.; Mazzoldi, A. Characterization and Modelling of a Conducting Polymer Muscle-like Linear Actuator. *Smart Mater. Struct.* **1997**, *6* (1), 23–34. https://doi.org/10.1088/0964-1726/6/1/003.

(25) Lu, W.; Fadeev, A. G.; Qi, B.; Smela, E.; Mattes, B. R.; Ding, J.; Spinks, G. M.; Mazurkiewicz, J.; Zhou, D.; Wallace, G. G.; MacFarlane, D. R.; Forsyth, S. A.; Forsyth, M. Use of Ionic Liquids for π-Conjugated Polymer Electrochemical Devices. *Science* **2002**, *297* (5583), 983–987. https://doi.org/10.1126/science.1072651.

(26) Spinks, G. M.; Liu, L.; Wallace, G. G.; Zhou, D. Strain Response from Polypyrrole Actuators under Load. *Adv. Funct. Mater.* **2002**, *12* (6–7), 437–440. https://doi.org/10.1002/1616-3028(20020618)12:6/7<437::AID-ADFM437>3.0.CO;2-I.
